# Neuropsychiatric and Laboratory Outcomes of Hepatitis C Treatment in an Early-Treated HIV Cohort in Thailand

**DOI:** 10.21203/rs.3.rs-4186965/v1

**Published:** 2024-04-03

**Authors:** Ferron F. Ocampo, Carlo Sacdalan, Suteeraporn Pinyakorn, Misti Paudel, Tanyaporn Wansom, Nathornsorn Poltubtim, Somchai Sriplienchan, Nittaya Phanuphak, Robert Paul, Denise Hsu, Donn Colby, Lydie Trautmann, Serena Spudich, Phillip Chan

**Affiliations:** SEARCH Research Foundation; SEARCH Research Foundation; Walter Reed Army Institute of Research; Walter Reed Army Institute of Research; Dreamlopments Foundation; SEARCH Research Foundation; SEARCH Research Foundation; Institute of HIV Research and Innovation; University of Missouri; Walter Reed Army Institute of Research; Walter Reed Army Institute of Research; Walter Reed Army Institute of Research; Yale University; Yale University

**Keywords:** HIV, hepatitis C, HIV/HCV coinfection, direct-acting antivirals, SVR, cognitive, PWH

## Abstract

**Background:**

Hepatitis C virus (HCV) coinfection may further compromise immunological and cognitive function in people with HIV (PWH). This study compared laboratory and neuropsychiatric measures across the periods of HCV seroconversion and direct-acting antiviral (DAA) therapy with sustained virologic response (SVR) among PWH who initiated antiretroviral therapy (ART) during acute HIV infection (AHI) and acquired HCV after 24 weeks of ART.

**Methods:**

Participants from the RV254 AHI cohort underwent paired laboratory and neuropsychiatric assessments during regular follow-up. The former included measurements of CD4 + and CD8 + T-cell counts, HIV RNA, liver enzymes, and lipid profiles. The latter included the Patient Health Questionnaire-9 (PHQ-9), Distress Thermometer (DT), and a 4-test cognitive battery that evaluated psychomotor speed, executive function, fine motor speed and dexterity. The raw scores in the battery were standardized and averaged to create an overall performance (NPZ-4) score. Parameters of HCV-coinfected participants were compared across HCV seroconversion and DAA treatment groups.

**Results:**

Between 2009 and 2022, 79 of 703 RV254 participants acquired HCV after ≥ 24 weeks of ART; 53 received DAA, and 50 (94%) achieved SVR. All participants were Thai males (median age: 30 years); 34 (68%) denied past intravenous drug use, and 41 (82%) had a history of other sexually transmitted infections during follow-up. Following SVR, aspartate transferase (AST) and alanine transaminase (ALT) decreased (p < 0.001), while total cholesterol, low-density lipoprotein, and triglycerides increased (p < 0.01). The median CD4+/CD8 + ratio increased from 0.91 to 0.97 (p = 0.012). NPZ-4 improved from 0.75 to 0.91 (p = 0.004). The median DT score increased from 1.7 to 2.7 (p = 0.045), but the PHQ-9 score remained unchanged.

**Conclusion:**

HCV coinfection is common in this group of high-risk PWH, highlighting the need for regular screening, early diagnosis, and treatment. There was a modest improvement in the CD4+/CD8 + T-cell ratio and cognitive performance after DAA therapy in patients who achieved SVR. Future studies should examine potential neuropsychiatric impacts during early HCV infection as well as the longer-term neuropsychiatric outcomes after DAA treatment with SVR.

## Background

HIV and Hepatitis C virus (HCV) share common modes of transmission, including sexual contact, needle-sharing during intravenous drug use (IVDU), and blood product transfusion. HCV coinfection is reported in up to 6.2% of people with HIV (PWH) [1]. The odds of HCV infection are 6 times greater among PWH than among individuals without. Compared to HIV monoinfection and HCV monoinfection, HIV/HCV coinfection is associated with greater risks of mortality, liver failure and extrahepatic manifestations, including cognitive and affective symptoms [2–6], indicating that disease progression is accelerated in individuals with dual infections.

The use of direct-acting antivirals (DAAs) averts the devastating consequences of chronic HCV infection, as evidenced by a high cure rate (sustained virologic response, SVR) and favorable safety profiles [7]. DAAs inhibit the nonstructural proteins that are responsible for viral replication. Remarkably, DAAs are effective regardless of age, sex, previous treatment experience, or stage of liver fibrosis [8]. Improvements in HCV-related extrahepatic conditions after DAA therapy and SVR [9] were observed in HCV-monoinfected as well as HIV/HCV-coinfected individuals [10].

To date, most HCV treatment outcome studies focusing on immunologic and neuropsychiatric outcomes in HIV/HCV-coinfected patients have been based on individuals with chronic HIV infection, heterogeneous HIV suppression status, and often, an unknown duration of HCV infection [7, 8]. Moreover, only a few of them provided a concurrent account of changes before and after HCV seroconversion prior to HCV treatment. This study examined the changes in laboratory and neuropsychiatric parameters during the periods of HCV seroconversion and DAA therapy with SVR among research participants from a Thai acute HIV infection (AHI) cohort who were on antiretroviral therapy (ART).

## Methods

### Study design and participants

The study participants were from the RV254 AHI cohort study in Bangkok, Thailand, which enrolled individuals with Fiebig I-V AHI [11]. RV254 participants typically commenced ART within a median of 3 days post enrollment and were longitudinally followed. They were screened for HCV infection every 48 weeks using an HCV-antibody test, and active infection was confirmed through HCV RNA measurement via the Xpert HCV Assay (Cepheid, Sunnyvale, CA). The study protocol was approved by the institutional review boards of all relevant collaborating institutions. All participants provided written informed consent.

### Participant selection

The analysis included participants who met the following criteria: (1) tested negative for HCV antibody at enrollment; (2) had ≥ 24 weeks of ART with suppressed HIV RNA levels (<50 copies/ml) before initiating DAA treatment; (3) achieved SVR with undetectable HCV RNA ≥ 12 weeks following a standard course of DAA treatment; and (4) completed paired laboratory and neuropsychiatric assessments before and after DAA therapy with SVR, based on the RV254 study protocol (see below).

### Laboratory and clinical investigations

Blood tests included HIV-related immunologic and virologic parameters (plasma HIV RNA, CD4 + and CD8 + T-cell counts), complete blood count, liver enzyme levels (aspartate transferase (AST) and alanine transaminase (ALT)) and lipid profile (total cholesterol, low-density lipoprotein (LDL) and triglyceride). Additionally, nontreponemal syphilis testing and nucleic acid amplification tests for chlamydia and gonorrhea are performed every 24 and 48 weeks, respectively. Participants are also screened for peripheral neuropathy by research clinicians every 48 weeks (**Supplementary Table 1**).

### Neuropsychiatric assessments

All RV254 participants underwent neuropsychiatric assessments at enrollment; at 12, 24 and 48 weeks; and every 48 weeks thereafter. Mood assessments included the Patient Health Questionnaire-9 (PHQ-9) and the Distress Thermometer (DT). Both have been validated for use in Thailand [12–14]. The PHQ-9 is a 9-item survey of depressive symptoms (score range 0–27) derived from the DSM-IV [15]. Total scores ≥ 10 and ≥ 15 indicate moderate and moderate-severe depression, respectively [15]. The DT is a self-reported measure of emotional distress that is analogous to a visual analog scale with a range from 0 to 10 [16].

Cognitive assessment was based on a 4-test battery that measured motor speed and dexterity (nondominant hand Grooved Pegboard test; Lafayette Instrument Company, Lafayette, IN, USA), psychomotor speed (Color Trails 1 and Trail Making A; PAR, Inc., Lutz, FL, USA), and executive functioning (Color Trails 2; PAR, Inc., Lutz, FL, USA). Raw scores were standardized to z-scores using Thai normative data [17], which were averaged to create an overall performance (NPZ-4) score. Individual and overall test performances were included in the analyses.

### Data analysis

The paired laboratory and neuropsychiatric measures of the participants with HCV were assessed across two separate periods, specifically in relation to HCV seroconversion and DAA therapy: (1) **Pre-HCV seroconversion:** last visit prior to HCV seroconversion versus **Post-HCV seroconversion:** first visit after HCV seroconversion; and (2) **Pre-DAA:** last visit before DAA initiation versus **Post-DAA:** first visit after completion of DAA therapy with SVR. Outcomes are reported as medians (interquartile ranges, IQRs) or frequencies (percentages) and were analyzed using McNemar’s test and the Wilcoxon signed-rank test in StataCorp. 2019. Stata Statistical Software: Release 16. College Station, TX: StataCorp LLC.

## Results

**Figure 1:**
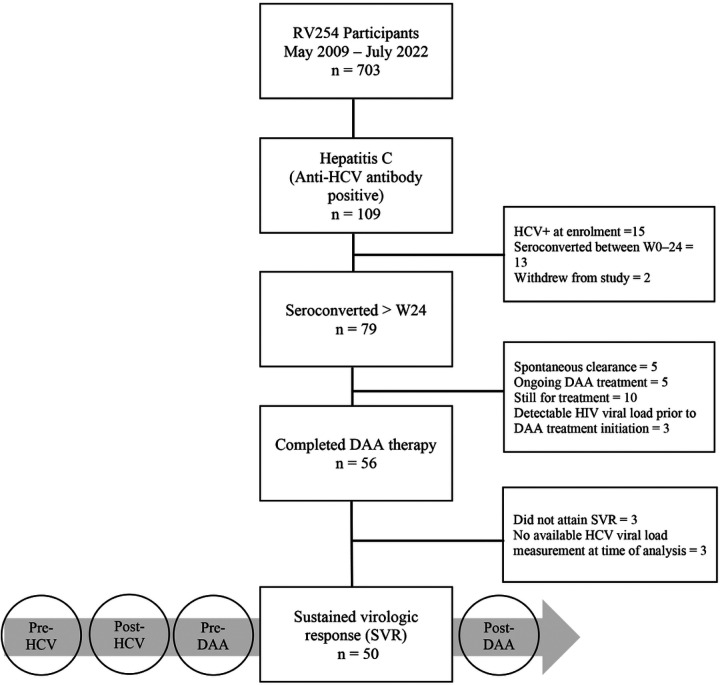
Study Participant Identification and Selection

Between May 2009 and July 2022, 109 (15.5%) out of 703 RV254 participants were diagnosed with HCV: 16 tested positive for anti-HCV antibody at enrollment (week 0), 12 seroconverted between weeks 1 and 24, and 79 were diagnosed after 24 weeks of ART; 2 withdrew from the study at the time of analysis. Among the 79 participants who seroconverted after 24 weeks of ART, 53 completed DAA therapy with HCV RNA level measurements at least 3 months after DAA therapy. Of these, 50 (94%) achieved SVR and were included in the current analysis (Fig. 1).

All 50 participants were Thai males with a median age of 30 [IQR 26–35] years. The median duration from RV254 enrollment to HCV seroconversion was 192 [IQR 96–318] weeks. Forty-three (86%) were infected with HCV genotype 1. Thirty-four (68%) denied any prior IVDU, while 41 (82%) contracted other sexually transmitted infections (STIs), including syphilis, gonorrhea, and chlamydia, during follow-up (Table 1). The median durations between HCV seroconversion and pre-DAA assessment and between DAA completion and post-DAA assessment were 15 [IQR 9, 29] and 27 [IQR 20–38] weeks, respectively. The predominant DAA regimens used were sofosbuvir/ledipasvir (52%) and sofosbuvir/velpatasvir (36%). None reported DAA-related adverse events.

### Laboratory Outcomes

Table 2 compares the outcome measures across the periods of HCV seroconversion (pre-HCV seroconversion vs. post-HCV seroconversion, median duration 49 IQR [47–91] weeks) and DAA treatment (pre-DAA vs. post-DAA and SVR, median duration 49 IQR [48–68] weeks). Notably, measures of 38 participants (76%) post-HCV seroconversion and pre-DAA originated from the same follow-up visit in 38 participants (76%). The levels of ALT (28.5 IQR [18.8, 48.3] vs. 98.5 IQR [49–180]) and AST (20.5 IQR [16–25.5] vs. 56 IQR [36–100]) increased after HCV seroconversion (p < 0.001) and subsequently decreased (ALT: 99 IQR [49–179] vs. 19 IQR [13–26], p < 0.001; AST: 56 IQR [36–100] vs. 20 IQR [16–25], p < 0.001) following DAA and SVR. The lipid parameters were not significantly different after HCV seroconversion. However, the levels of total cholesterol (184 IQR [159–210] vs. 201 IQR [174–225]), LDL (113 IQR [94–138] vs. 136 IQR [111–160]), and triglycerides (87 IQR [72–119] vs. 112 IQR [82–161]) increased after DAA treatment in patients who achieved SVR (p < 0.001). The levels of hemoglobin, total white blood cell count, and platelet count remained statistically unchanged across all timepoints.

The CD4+ (639 IQR [496–768] vs. 687 IQR [569.50–815], p = 0.03) and CD8+ (669 IQR [550–852] vs. 776 IQR [586–912], p = 0.03) T-cell counts increased after HCV seroconversion and exhibited an insignificant decrease after DAA treatment and SVR. On the other hand, the median CD4+/CD8 + T-cell ratio was not significantly different after HCV seroconversion but increased from 0.91 [IQR 0.73–1.1] to 0.97 [IQR 0.76–1.29] (p = 0.012) after DAA treatment and SVR. The plasma HIV suppression rate remained unchanged throughout the two periods.

### Neurological and Neuropsychiatric Outcomes

None of the 50 participants exhibited signs of peripheral neuropathy before or immediately after seroconversion, but two (4%) demonstrated signs of peripheral neuropathy prior to the initiation of DAA treatment. One participant (2%) demonstrated persistent signs of peripheral neuropathy after DAA therapy and SVR. Cognitive test performance, including the composite NPZ-4 and z-scores in each of the 4 tests, was statistically unchanged after HCV seroconversion. Following DAA treatment and SVR, NPZ-4 modestly improved from 0.75 [IQR 0.42–1.02] to 0.91 [IQR 0.55–1.31] (p = 0.004). The change was accompanied by an improvement in the median z-scores of all four cognitive tests. However, only the improvement in the Trail Making A test score was statistically significant (0.60 [IQR 0.17–1.32] vs. 0.91 [IQR 0.4–1.4], p = 0.028). The median DT and PHQ-9 scores remained similar before and after HCV seroconversion. After DAA therapy and SVR, the median DT score increased from 1.7 [IQR 1–4.2] to 2.7 [IQR 1.1–5] (p = 0.045). The PHQ-9 total score and the frequencies of moderate and moderate-severe depression remained statistically unchanged.

## Discussion

We previously reported an HCV epidemic among HIV-infected men who have sex with men (MSM) in Bangkok, Thailand [18, 19]. We observed a 16% prevalence of HCV infection in this young, MSM-predominant AHI cohort, with an 11% cumulative incidence of HCV seroconversion among participants on stable (> 24 weeks) ART. In comparison, a global systematic study reported that the prevalence of HCV coinfection in HIV-infected MSM is approximately 6.4% [1]. Notably, the main route of HCV transmission in our cohort was likely through sexual behavior, primarily anal sex, often in the context of substance use and group sex [18]. While 32% of the study participants reported a history of IVDU, problematic substance use and substance dependence were rare, as indicated by clinician interviews during follow-up visits.

Consistent with the high efficacy and safety profile of DAAs [7], the treatment success rate was 94%, and DAA-related adverse events were not observed in this study. The levels of liver enzymes decreased post-DAA, re ecting the resolution of HCV-related hepatic in ammation. Previous studies reported increases in hemoglobin levels, total leukocyte counts, and platelet counts after SVR [20–22], but these changes were not observed here. The hematological benefits reported in prior studies could be secondary to improvements in liver fibrosis, portal hypertension, and anemia associated with chronic illness after SVR [23]. These hematological changes were not observed in our cohort, most likely because our participants were relatively young MSM who, prior to DAA initiation, had normal hematological parameters and lacked major hepatic complications. In line with the results of prior studies that included HCV-monoinfected [24–26] and HIV/HCV-coinfected participants [27], we noted elevations in total cholesterol, triglyceride, and LDL levels after SVR. HCV is hypothesized to induce hypolipidemia by stimulating LDL receptor expression and modulating proteins involved in liver steatosis [27, 28]. HCV eradication may therefore reverse these processes, leading to paradoxical increases in serum lipid levels.

### HIV-related immune and virologic parameters

HCV infection has been reported to negatively impact the ART response and CD4 + T-cell recovery in ART-naïve PWH [29]. HCV infection induces CD8 + T-cell activation and may hamper CD8 + T-cell downregulation in PWH on ART [30], thereby worsening CD4+/CD8 + T-cell ratio inversion [31]. Two previous studies reported unchanged CD4 + T-cell counts post-SVR [32, 33]. While two studies reported a reduction in the CD8 + T-cell count post-SVR [32, 34], only one reported a concomitant improvement in the CD4+/CD8 + T-cell ratio [34]. In this study, the CD4+/CD8 + T-cell ratio modestly increased without statistically significant changes in CD4 + and CD8 + T-cell counts in the context of stable HIV suppression between pre-DAA and post-DAA visits. Nonetheless, in the absence of a comparison group of HIV-monoinfected and HCV-monoinfected individuals, it is challenging to disentangle the effects of DAA treatment versus antiretroviral therapy on CD4+/CD8 + T-cell recovery.

### Neurological and Neuropsychiatric Outcomes

Neurological complications, such as cognitive impairment, mood disorders, and peripheral neuropathy, have been reported in individuals with HCV monoinfection and HIV/HCV coinfection [35–38]. In this study, peripheral neuropathy was uncommon and was identified in 2 participants (4%) who exhibited mild impairment of distal vibration sense within six months of HCV seroconversion. The neuropathy symptoms were likely HCV-related given their temporal relationship, stable HIV suppression, and normal folate, vitamin B12, and glycosylated hemoglobin (HbA1c) levels. In an HCV monoinfection study, neuropathy symptoms improved in half of the participants after DAA therapy [39], whereas one participant in our study experienced resolved neuropathy symptoms nine months after DAA therapy.

Surprisingly, DT scores increased post-DAA in this study compared to a reduction in a prospective study with 90 HCV-monoinfected participants [40]. Nonetheless, the median DT score at the post-DAA visit remained well below the commonly referred cutoff score (≥ 4), which signifies distress with clinical significance [41]. Notably, this change was not associated with worsening of the PHQ-9 score.

HCV infection might impact cognition [42, 43] through direct neurotoxicity, neuroin ammation, and hepatic encephalopathy [42–44]. HCV/HIV coinfection has been associated with worse cognition than either HIV or HCV monoinfection [45–47]. Most studies have shown significant cognitive improvement post-DAA with SVR, especially in visual learning/memory, executive functions, verbal uency, processing speed, and motor skills [47–49]. In our study, both the composite score and z-score of the Trail Making A test (a test of psychomotor speed) improved post-DAA. Notably, both indices were within the normal range before DAA, and the magnitude of improvement was modest and within the standard error of measurement.

To our knowledge, this is the first study that concurrently offers a longitudinal assessment of laboratory, cognitive and mood outcomes in PWH throughout the phases of HCV seroconversion and DAA treatment. The analysis was based on a longitudinal cohort of PWH who initiated ART during acute HIV and acquired HCV while on stable ART for more than 24 weeks. The present study design has partially addressed several methodological confounders of previous studies on cognitive outcomes after DAA treatment and HCV eradication. First, practice effects lead to improved performance on several NP measures, especially between the first and second test exposures [50]. In this study, participants completed the same cognitive battery multiple times and passed the critical phase of practice effects before DAA therapy was initiated, limiting the impact on test performance. Second, disentangling the potential cognitive benefits of HCV eradication from those of ART on HIV infection is challenging, as cognitive improvement related to the latter may take weeks to months before reaching a plateau [51]. Similarly, CD4 + and CD8 + T-cell counts continuously evolve months to years after ART initiation [52]. In this study, the potential immunological and cognitive benefits of ART were minimized by the stable use of ART for more than 2 years before initiating DAA treatment in most of the participants. The initially statistically unchanged NPZ-4 and CD4+/CD8 + scores after HCV seroconversion and the subsequent improvement in these parameters after DAA treatment with SVR raise the question of whether the observed improvement is related to DAA treatment and HCV eradication.

Limitations of this study include the sample size, short follow-up duration, male-only setting, the absence of liver elastography to grade the severity of liver disease, and the lack of HCV-monoinfected controls to determine whether the observed changes are HIV/HCV-coinfection specific.

## Conclusion

Consistent with existing reports, this study highlighted the high frequency of HCV coinfection among young MSM with HIV in our locality. These patients responded well to DAA treatment, achieving a high rate of HCV eradication. These findings indicate the necessity of incorporating routine HCV screening for early diagnosis, along with educational materials on HCV prevention, into ongoing HIV/AIDS programs. We observed modest longitudinal improvements in cognitive test performance and the CD4+/CD8 + T-cell ratio after DAA therapy and SVR but not during the period after HCV seroconversion. The discordant outcomes between the two periods may suggest an early neuropsychiatric impact of HCV infection, which was subsequently reversed by prompt initiation of HCV treatment and eradication. Future studies with both HIV- and HCV-monoinfected controls should focus on the early neuropsychiatric impacts of HCV infection as well as the longer-term neuropsychiatric outcomes after DAA treatment with SVR.

## Figures and Tables

**Table 1.** Participants’ characteristics^1^ (N=50)

Age, years	30 (26, 35)
Male sex, n (%)	50 (100)
Weight, kg	62.8 (54.9, 71.4)
Body mass index, kg/m^2^	21.4 (19.08, 24.1)
Duration from acute HIV to HCV seroconversion, weeks	192 (96, 318)
Plasma HIV RNA at HCV seroconversion, log_10_ cps/ml	1.3 (1.3, 1.3)
Plasma HCV RNA at HCV seroconversion, log_10_ cps/ml	5.90 (4.84, 6.76)
**HCV Genotype, n (%)**	
1	43 (86)
3	2 (4)
Not determined	5 (10)
**Sexual Orientation, n (%)**	
MSM	
Homosexual	47 (94)
Bisexual	3 (6)
Heterosexual	0 (0)
**Substance use, n (%)**	
Intravenous	16 (32)
Oral/Inhalational	14 (28)
**Incident Sexually transmitted infection, n (%)**	
Syphilis	37 (74)
Gonorrhea	41 (82)
Chlamydia	35 (70)
**Hepatitis B co-infection (+HBs antigen), n (%)**	0 (0)
**Transfusion products, n (%)**	0 (0)
Duration between HCV diagnosis and DAA initiation, weeks	55 (29, 69)
**ART Regimen prior to HCV seroconversion, n (%)**	
Dolutegravir-based	44 (88)
Efavirenz-based	6 (12)
**DAA Regimens, n (%)**	
Sofosbuvir/Ledipasvir	26 (52)
Sofosbuvir/Velpatasvir	18 (36)
Sofosbuvir/Daclatasvir	3 (6)
Sofosbuvir/Ravidasvir	3 (6)

1 Median (IQR) is provided unless specified

**Table 2:** Laboratory and clinical outcomes across HCV seroconversion and DAA treatment with SVR

Parameters	Pre-HCV Seroconversion^1^	Post-HCV Seroconversion^2^	p-value	Pre-DAA^3^	Post-DPA^4^	p-value
*Biochemistry and Hematologic*						
ALT, U/L	28.5 (18.8, 48.3)	98.5 (49,180)	**<0.001**	99 (49, 179)	19 (13, 26)	**<0.001**
AST^5^, U/L	20.5 (16, 25.5)	56 (36, 100)	**<0.001**	56 (36, 100)	20 (16, 25)	**<0.001**
Plasma HCV RNA, log_10_ cps/mL	0	6.35 (5.33, 6.91)	**<0.001**	6.35 (5.33, 6.91)	<1.08 (1.08, 1.08)	**<0.001**
Total white blood cell count, 10^9^/L	6.09 (5.12, 7.05)	5.99 (4.85, 6.95)	0.19	5.99 (4.86, 6.94)	5.8 (4.92, 6.88)	0.96
Platelet count, 10^9^/L	265 (241, 306)	269 (245, 298)	0.95	269 (246, 298)	277 (246, 304)	0.57
Hemoglobin, g/dL	14.8 (14.3, 15.4)	15.1 (14.3, 15.5)	0.06	15.1 (14.3, 15.5)	14.9 (13.7, 15.6)	0.15
Total cholesterol, mg/dL	185 (158, 212)	184 (159, 210)	0.48	184 (159, 210)	201 (174, 225)	**<0.001**
Low density lipoprotein (LDL), mg/dL	121 (96, 138)	113 (94, 140)	0.66	113 (94, 138)	136 (111, 160)	**<0.001**
Triglycerides, mg/dL	86 (64, 135)	87 (72, 121)	0.53	87 (72, 119)	112 (82, 161)	**0.003**
*Immunologic and Virologic*						
CD4 + T-cell count, cells/mm^3^	639 (496, 768)	687 (569.50, 815)	**0.03**	687 (571, 813)	660 (513, 925)	0.99
CD8 + T-cell count, cells/mm^3^	669 (550, 852)	776 (586, 912)	**0.03**	776 (588, 909)	736 (542, 863)	0.17
CD4/CD8 ratio	0.90 (0.77, 1.14)	0.91 (0.73,1.10)	1.00	0.91 (0.73, 1.1)	0.97 (0.76, 1.29)	**0.012**
HIV RNA< 50 copies/ml, n (%)	49 (98)	50 (100)	0.60	50 (100)	50 (100)	1.00
*Peripheral neuropathy, n (%)*	0 (0)	0 (0)	1.00	2 (4)	1 (2)	1.00
*Cognitive Tests*						
NPZ-4 score	0.72 (0.30, 1.15)	0.75 (0.40, 1.02)	0.65	0.75 (0.42, 1.02)	0.91 (0.55, 1.31)	**0.004**
z-Color Trails 1	1.30 (0.70, 1.62)	1.23 (0.70, 1.66)	0.63	1.23 (0.74, 1.65)	1.29 (0.84, 1.73)	0.29
z-Color Trails 2	0.59 (−0.10, 1.16)	0.78 (0.21, 1.48)	0.06	0.78 (0.21, 1.46)	0.92 (0.44, 1.26)	0.52
z-Grooved Pegboard	0.62 (−0.10, 1.38)	0.43 (−0.49, 1.22)	0.10	0.43 (−0.49, 1.19)	0.79 (−0.05, 1.38)	0.08
z-Trails Making A	0.85 (0.15, 1.19)	0.60 (0.13, 1.33)	0.18	0.60 (0.17, 1.32)	0.91 (0.4, 14)	**0.03**
*Mood Assessments*						
PHQ-9 total score (range 0–27)	6 (2,9)	6 (2, 8)	0.58	6 (2, 8)	4 (2, 8)	0.72
PHQ-9 ≥10, n (%)	5 (10)	7 (14)	0.38	11 (22)	9 (18)	0.73
PHQ-9 ≥15, n (%)	3 (6)	4 (8)	0.70	4 (8)	3 (6)	1
Distress Thermometer (range 0–10)	2 (0.9, 4.0)	1.7 (1, 4.2)	0.25	1.7 (1, 4.2)	2.7 (1.1, 5)	**0.05**

1 Last visit prior to HCV seroconversion

2 First visit after HCV seroconversion

3 Last visit prior to initiation of DAA treatment; Median of 15 weeks (IQR 9–29) before DAA

4 First visit after completion of DAA treatment and SVR; Median of 27 weeks (IQR 20–38) after DAA

5 n=34

## Data Availability

The data that support the findings of this study are available upon request from the corresponding author. The data are not publicly available due to privacy or ethical restrictions.

## List Of Abbreviations

ART: antiretroviral therapy
DAA: direct-acting antiviral
CT1: Color Trails 1
CT2: Color Trails 2
DT: Distress Thermometer
GPB: Grooved Pegboard
HIV: human immunodeficiency virus (HIV)
HCV: hepatitis C virus
IVDU: intravenous drug use
PWH: people with HIV
STI: sexually transmitted infection
SVR: sustained virologic response
TMA: Trails Making A
